# Circular spectropolarimetric sensing of higher plant and algal chloroplast structural variations

**DOI:** 10.1007/s11120-018-0572-2

**Published:** 2018-08-23

**Authors:** C. H. Lucas Patty, Freek Ariese, Wybren Jan Buma, Inge Loes ten Kate, Rob J. M. van Spanning, Frans Snik

**Affiliations:** 1 Molecular Cell Physiology, VU Amsterdam, De Boelelaan 1108, 1081 HZ Amsterdam, The Netherlands; 2 LaserLaB, VU Amsterdam, De Boelelaan 1083, 1081 HV Amsterdam, The Netherlands; 3 HIMS, Photonics Group, University of Amsterdam, Science Park 904, 1098 XH Amsterdam, The Netherlands; 4 Department of Earth Sciences, Utrecht University, Budapestlaan 4, 3584 CD Utrecht, The Netherlands; 5 Systems Bioinformatics, VU Amsterdam, De Boelelaan 1108, 1081 HZ Amsterdam, The Netherlands; 6 Leiden Observatory, Leiden University, P.O. Box 9513, 2300 RA Leiden, The Netherlands

**Keywords:** Circular polarization, Photosynthesis, Chloroplast, Chlorophyll, Algae

## Abstract

Photosynthetic eukaryotes show a remarkable variability in photosynthesis, including large differences in light-harvesting proteins and pigment composition. In vivo circular spectropolarimetry enables us to probe the molecular architecture of photosynthesis in a non-invasive and non-destructive way and, as such, can offer a wealth of physiological and structural information. In the present study, we have measured the circular polarizance of several multicellular green, red, and brown algae and higher plants, which show large variations in circular spectropolarimetric signals with differences in both spectral shape and magnitude. Many of the algae display spectral characteristics not previously reported, indicating a larger variation in molecular organization than previously assumed. As the strengths of these signals vary by three orders of magnitude, these results also have important implications in terms of detectability for the use of circular polarization as a signature of life.

## Introduction

Terrestrial biochemistry is based upon chiral molecules. In their most simple form, these molecules can occur in a left-handed and a right-handed version called enantiomers. Unlike abiotic systems, nature almost exclusively uses these molecules in only one configuration. Amino acids, for instance, primarily occur in the left-handed configuration while most sugars occur in the right-handed configuration. This exclusive use of one set of chiral molecules over the other, called homochirality, therefore serves as a unique and unambiguous biosignature (Schwieterman et al. 2018).

Many larger, more complex biomolecules and biomolecular architectures are chiral too and the structure and functioning of biological systems is largely determined by their chiral constituents. Homochirality is required for processes ranging from self-replication to enzymatic functioning and is therefore also deeply interwoven with the origins of life.

The phenomenon of chirality, i.e., the molecular dissymmetry of chiral molecules, causes a specific response to light (Fasman 2013; Patty et al. 2018a). This response is both dependent on the intrinsic chirality of the molecular building blocks and on the chirality of the supramolecular architecture. Polarization spectroscopy enables these molecular properties to be probed non-invasively from afar and is therefore of great value for astrobiology and the search for life outside our solar system. Polarization spectroscopy also has a long history in biological and chemical sciences. Circular dichroism (CD) spectroscopy utilizes the differential electronic absorption response of chiral molecules to left- and right-handed circularly polarized incident light and is very informative for structural and conformational molecular dynamics. As such it has proven to be an indispensable tool in (bio-)molecular research.

Chirality can also be observed in chlorophylls and bacteriochlorophylls utilized in photosynthesis. While their intrinsic CD signal is very weak due to their almost planar symmetrical structure, these chlorophylls are organized in a chiral supramolecular structure that greatly enhances these signals (Garab and van Amerongen 2009). This is particularly the case for the photosynthetic machinery in certain eukaryotes, where photosynthesis is carried out in specialized organelles, chloroplasts, which in higher plants have a large molecular density yielding anomalously large signals: polymer- and salt-induced (psi)-type circular dichroism (Keller and Bustamante 1986; Garab and van Amerongen 2009; Garab et al. 1991a; Tinoco et al. 1987).

While circular dichroism spectroscopy depends on the modulation of incident light to detect the differential extinction of circularly polarized light, we have recently shown that in leaves comparable results can be obtained by measuring the induced fractional circular polarization of unpolarized incident light (Patty et al. 2017, 2018b). As the latter only requires modulation in front of the detector it offers unique possibilities, allowing to probe the molecular architecture from afar. In vegetation, the influence of photosynthesis functioning and vegetation physiology on the polarizance could provide valuable information in Earth remote sensing applications, as was demonstrated for decaying leaves (Patty et al. 2017). As homochirality is a prerequisite for these signals (left- and right-handed molecules display an exactly opposite signal and will thus cancel out each other if present in equal numbers) and is unique to nature, circular polarization could also indicate the unambiguous presence of life beyond Earth and as such is a potentially very powerful biosignature (Sparks et al. 2009a, b; Wolstencroft 1974; Patty et al. 2018a; Pospergelis 1969; Schwieterman et al. 2018).

Higher plants evolved relatively recently in contrast to microbial life. Biosignatures of microbial life are mostly focused on astrobiology [and which also display typical circular polarization signals (Sparks et al. 2009a)]. While molecular analysis suggests higher plants appeared by 700 Ma (Heckman et al. 2001), the earliest fossil records date back to the middle Ordovician ($\sim$ 470 Ma) (Wellman and Gray 2000). The earliest microbial fossil records date back to 3.7 Ga (Nutman et al. 2016) and oxygenic photosynthesis (in cyanobacteria) is likely to have evolved before 2.95 Ga (Planavsky et al. 2014). It is however unclear if photosynthetic microbial life would be able to colonize terrestrial niches extensively enough to be used as a remotely detectable biosignature.

**Fig. 1:**
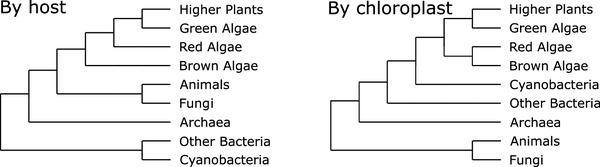
Evolutionary relationships based on the host rRNA (left) and based on chloroplast DNA (cpDNA) (right)

On the other hand, these photosynthetic bacteria stood at the basis of the evolution of higher plants as their photosynthetic apparatus evolved from a endosymbiosis between a cyanobacterium and a heterotrophic host cell. It is widely accepted that all chloroplasts stem from a single primary endosymbiotic event (Moreira et al. 2000; Ponce-Toledo et al. 2017; McFadden 2001). Not all photosynthetic eukaryotes, however, descend from this endosymbiotic host, as certain algae acquired photosynthesis through secondary endosymbiosis of a photosynthetic eukaryote (McFadden 2001; Green 2011). The simplified evolutionary relations between the different algae, based on the host and on the chloroplasts, are shown in Fig. 1.

**Fig. 2:**
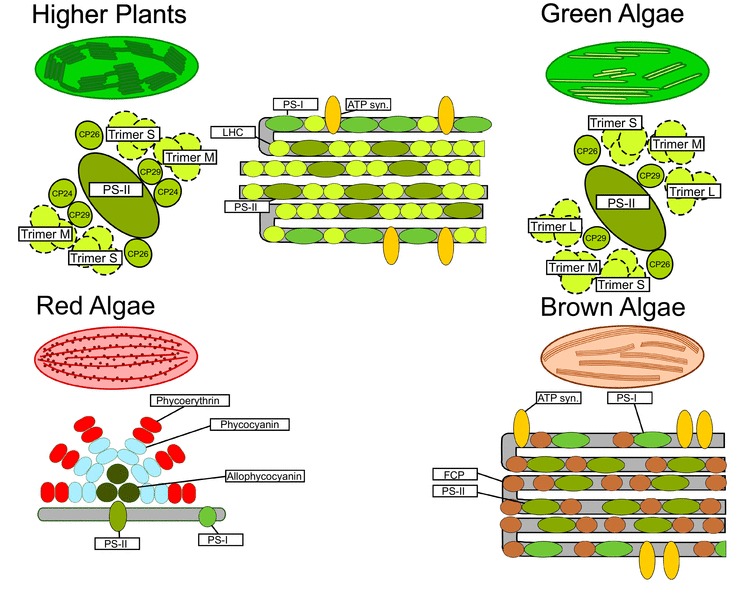
Schematic representation of the photosynthetic structures of higher plants and algae. There is a distinct organizational difference in the supercomplexes between higher plants and algae. Additionally, while green algae display stacked thylakoid membranes, they lack true grana. Red algae contain phycobilisomes, unlike the other algae. In brown algae the thylakoid membranes are threefold and the supercomplex organization is not entirely resolved

Although algae contribute up to 40% of the global photosynthesis (Andersen 1992), they have received limited attention in astrobiology so far. While not as ancient as microbial life, algae are considerably older than plants, with fossil evidence of red algae dating back to 1.6 Ga (Bengtson et al. 2017). Additionally, molecular research on algae has mainly focused on a few unicellular algae, rather than multicellular species, and systematic studies on the chiral macro-organization of algal photosynthesis are lacking (Garab and van Amerongen 2009). Despite the common origin, millions of years of evolution has caused chloroplasts to show a remarkable diversity and flexibility in terms of structure (Fig. 2).

In higher plants, the chloroplasts typically display cylindrical grana stacks of 10–20 membrane layers that have a diameter of 300–600 nm. The stacks are interconnected by lamellae of several hundred nm in length (Mustárdy and Garab 2003). Additionally, certain plants can display grana stacks of more than 100 membrane layers (Anderson et al. 1973, Steinmann and Sjöstrand 1955) while the bundle sheath cells of certain C4 plants, such as maize, lack stacked grana and only contain unstacked stroma lamellae (Faludi-Daniel et al. 1973).

In higher plants, the psi-type circular polarizance is largely dependent on the size of the macrodomains formed by the photosystem II light-harvesting complex II supercomplexes (PSII–LHCII). The structure of PSII–LHCII in higher plants is relatively well known and consists of a dimeric PSII core complex C_2_ and associated trimeric LHCII, subdivided in three types based on their position and association with the core: Loose (L), Moderate (M), and Strong (S). Additionally, three minor antennae occur as monomers (CP24, CP26, CP29) (Boekema et al. 1999). The position of trimer L is still unclear and has so far only been observed in spinach (Boekema et al. 1999). The protein constituents and their typical circular polarization signature have been determined by Tóth et al. (2016). Furthermore, the negative band of the psi-type split signal is associated with the stacking of the thylakoid membranes, whereas the positive band is associated with the lateral organization of the chiral domains (Garab et al. 1988a, 1991b; Cseh et al. 2000).

The evolutionary history of grana and their functional advantage has been a matter of debate. It has been proposed that the structural segregation by grana of PSII and PSI prevents excitation transfer between these systems (Albertsson 2001; Nevo et al. 2012; Trissl and Wilhelm 1993). The extended compartmentation brought upon by grana might also aid regulatory pathways such as used in carbon fixation (Anderson 1999). It has been suggested that grana facilitates the regulation of light harvesting and enhance PSII functioning from limiting to saturating light levels, while at the same time protecting it from sustained high irradiance (Anderson 1999). Together with other adaptations, it has been hypothesized that these changes might have ultimately enabled green algae/plants to colonize and dominate various terrestrial niches (Nevo et al. 2012). Others have suggested that it might simply be a lack of competition; red algae for instance have probably experienced several evolutionary bottlenecks, vastly decreasing their genome size and therewith their potential for evolutionary adaptation (Collen et al. 2013).

Most closely related to higher plants are the green algae, which share a quite recent common ancestor. Similar to higher plants, green algae contain chlorophyll *a* and *b*. The structural composition of their photosynthetic machinery and the associated genes is primarily known from the unicellular green algae *Chlamydomonas*. Despite the high sequence similarity there are significant differences between the supercomplexes of higher plants and green algae. Importantly, green algae lack CP24, resulting in a different organization of the PSII–LHCII supercomplex (Tokutsu et al. 2012). While many green algae display thylakoid stacking, which can be up to seven membrane layers thick (Remias et al. 2005), true grana in green algae are rare and only occur in the late branching taxa Coleochaetales and Charales (Gunning and Schwartz 1999; Larkum and Vesk 2003).

Red algae also contain thylakoid membranes but these are never stacked. Furthermore, unlike green algae and plants, red algae can contain chlorophyll *d*, a pigment with an absorption band from 700 to 730 nm (Larkum and Kühl 2005). The red algae also contain phycobilisomes that serve as the primary antennae for PSII rather than the chlorophyll binding proteins found in higher plants and other algae. These phycobilisomes are homologous to those in cyanobacteria, but are lacking in plants and other algae (McFadden 2001).

Similarly, brown algae do not possess stacked thylakoid membranes but also do not contain phycobilins. All brown algae contain chlorophyll *a* and usually chlorophyll C_1_, C_2_, and/or C_3_. The light-harvesting systems in brown algae are based on fucoxanthin chlorophyll a/c_{1,2,3}_ proteins (FCP), which are homologous to LHC in higher plants/green algae but have a different pigment composition and organization (Premvardhan et al. 2010; Büchel 2015). Although this is still under debate (Burki et al. 2016), the brown algae have been classified as one supergroup (Dorrell and Smith 2011). Most brown algae have chloroplasts which were acquired through one or more endosymbiotic events with red algae (Dorrell and Smith 2011). Additionally, certain species of brown algae have been shown to display psi-type circular polarizance, although varying magnitudes of these signals have been reported, ranging from very weak to signals similar to higher plants [see (Garab and van Amerongen 2009) and references therein].

In the present study, we measure the fractional circular polarizance of various higher plants and multicellular algae. As the level of chiral macro-organization varies greatly between unicellular algae, we expect especially in multicellular algae that the organization can reach a higher or different level of complexity. These studies will additionally assess the feasibility of biosignature detection for (eukaryotic) photosynthesis from different evolutionary stages. While transmission and reflectance generally show a comparable spectral profile, the signals in reflectance are often weaker (e.g., due to surface glint). In the present study, we will therefore only display the results in transmission, as it provides better sensitivity for small spectral changes between samples.

## Materials and methods

### Sample collection

*Ulva lactuca*,* Porphyra* sp., and *Saccharina latissima* were grown in April at the Royal Netherlands Institute for Sea Research (NIOZ), using natural light and seawater. The algae were transported and stored in seawater at room temperature. Measurements on the algae were carried out within 2 days after acquisition.

*Ulva* sp., *Undaria pinnatifida*,* Grateloupia turuturu*, *S. latissima*, *Fucus serratus*, and *Fucus spiralis* were collected by Guido Krijger from WildWier^1^ from the North Sea near Middelburg in February. The algae were transported under refrigeration and stored in seawater. Measurements on the algae were carried out within 2 days after acquisition.

Leaves of *Skimmia japonica* and *Prunus laurocerasus* were collected in January from a private backyard garden near the city center of Amsterdam, *Aspidistra elatior* was obtained from the Hortus Botanicus Vrije Universiteit Amsterdam in February.

### Spectropolarimetry

For all measurements, three different samples were used (*n* = 3) and each single measurement is the average of at least 20,000 repetitions. Before each measurement, the samples were padded with paper towels to remove excess surface water. Circular polarization measurements were carried out in transmission and were performed using TreePol. TreePol is a dedicated spectropolarimetric instrument developed by the Astronomical Instrumentation Group at the Leiden Observatory (Leiden University). The instrument was specifically developed to measure the fractional circular polarization (*V*/*I*) of a sample interacting with unpolarized light as a function of wavelength (400–900 nm) and is capable of fast measurements with a sensitivity of ∼ 1 × 10^−4^. TreePol applies spectral multiplexing with the implementation of a dual fiber-fed spectrometer using ferro-liquid-crystal (FLC) modulation synchronized with fast read-out of the one-dimensional detector in each spectrograph, in combination with a dual-beam approach in which a polarizing beam splitter feeds the two spectrographs with orthogonally polarized light [see also (Patty et al. 2017)].

In this study, we have measured the induced fractional circular polarizance normalized by the total transmitted light intensity (*V*/*I*). Circular dichroism measures the differential absorption of left- or right-handed circularly polarized incident light, which is often reported in degrees *θ*. Under certain conditions, these two can be related and can therefore be converted by $V/I\approx \frac{2\pi \theta _{deg}}{180}$ [see also (Patty et al. 2018a)]. It has been shown that for leaves in transmission, the induced polarizance and the differential absorbance are comparable (Patty et al. 2017; 2018b), but we have not verified this for the samples used in this study.

## Results

### Higher plants

**Fig. 3:**
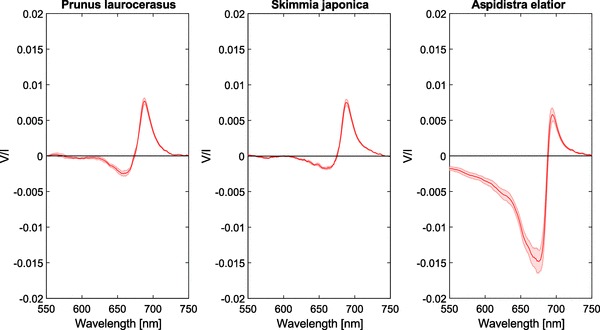
Circular polarimetric spectra of *S. japonica*, *P. laurocerasus*, and *A. elatior* leaves. Shaded areas denote the standard error, *n* = 3 per species

The circular polarization spectra of three different higher plants are shown in Fig. 3. For all species, we observe the typical split signal around the chlorophyll *a* absorption band ($\approx$ 680 nm) with a negative band at $\approx$ 660 nm and a positive band at $\approx$ 690 nm. The spectra of *Skimmia* and *Prunus* are very similar to each other in both shape and magnitude and show no significant differences. These results are also very similar to the results obtained for most other higher plants (data not shown). Interestingly, the circular polarimetric spectrum of *A. elatior* shows an exceedingly large negative band (−1.5 × 10^−2^) with a noticeable negative circular polarization extending much further into the blue, beyond the chlorophyll *a* (but also *b*) absorption bands. The positive band, however, has a similar magnitude (+ 6 × 10^−3^) as the other two plant species.

### Green algae

**Fig. 4:**
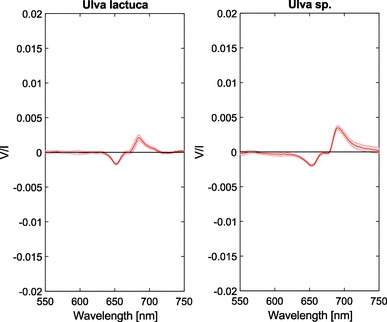
Circular polarimetric spectra of *U. lactuca* and *Ulva* sp. green algae. Shaded areas denote the standard error, *n* = 3 per species

The circular polarization spectra of two different green algae are shown in Fig. 4. Similar to higher plants, a split signal is observed around the chlorophyll *a* absorption band ($\approx$ 680 nm). Unlike higher plants, however, the negative and positive bands do not seem to overlap. The negative band reaches a *V*/*I* minimum at $\approx$ 655 nm and the positive band reaches a maximum at $\approx$ 690 nm, but the *V*/*I* signal is close to 0, and thus shows no net circular polarization between $\approx$ 665 to 678 nm. Additionally, the magnitude of the signals is much smaller than that of higher plants.

### Red algae

**Fig. 5:**
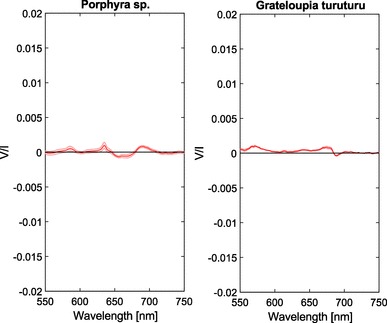
Circular polarimetric spectra of *Porphyra* sp. and *G. turuturu* red algae. Shaded areas denote the standard error, n = 3 per species

We show the circular polarization spectra of two different red algae in Fig. 5. These spectra show distinct differences compared to the higher plants and the green or brown algae. *Porphyra* sp. shows a continuous split signal around $\approx$ 680 nm, and an additional sharp positive feature at $\approx$ 635 nm. *G. turuturu* lacks these features and shows an inverse split signal around $\approx$ 680 nm. In both species, non-zero circular polarization can also be observed between 550 and 600 nm. We will further interpret these results in the Discussion.

### Brown algae

**Fig. 6:**
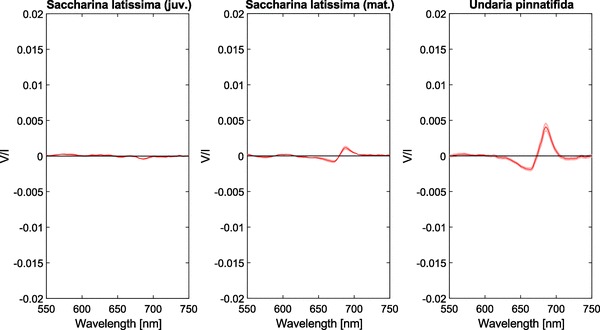
Circular polarimetric spectra of *S. latissima* (juvenile and mature) and *U. pinnatifida* brown algae. Shaded areas denote the standard error, *n* = 3 per species

**Fig. 7:**
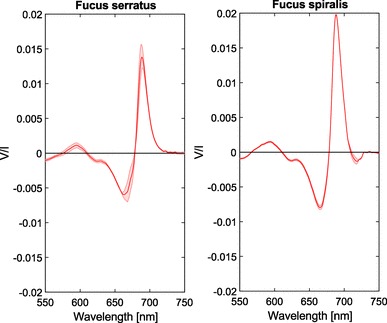
Circular polarimetric spectra of *F. serratus* and *F. spiralis* brown algae. Shaded areas denote the standard error, *n* = 3 per species

The brown algae exhibit a lot of variation in signal strength. For ease of comparison, the results of our circular spectropolarimetric measurements are plotted in Figs. 6 and 7 on the same y-scale. Figure 6 makes clear that a juvenile *S. latissima* barely displays a significant signal with the exception of a very weak negative feature (*V*/*I* = −4 × 10^−4^). The mature *S. latissima* samples show somewhat stronger bands, although the signal is still relatively small (−1 × 10^−3^, + 1 × 10^−3^). The polarimetric spectra of the brown algae *U. pinnatifida*, display a larger signal comparable to that of higher vegetation.

Interestingly, the polarimetric spectra of the brown algae of the genus *Fucus* display very large circular polarization signals, see Fig. 7. The alga *Fucus spiralis* has a *V*/*I* minimum and maximum of − 8 × 10^−3^ and + 2 × 10^−2^, respectively. Additionally, the bands are relatively narrow, with less polarization outside the chlorophyll *a* absorbance band. In the polarimetric spectra of *F. spiralis*, and to a lesser extent also of *U. pinnatifida*, a small negative band can be observed at 720 nm. Additionally, in the spectra of both *F. serratus* and *F. spiralis*, a positive band can be observed at 595 nm.

### *V*/*I* versus absorbance

**Fig. 8:**
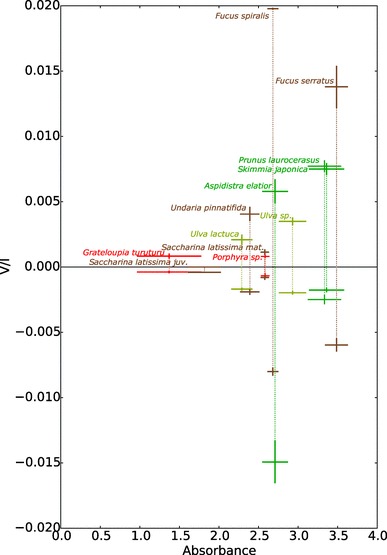
Maximum extend of the *V/I* bands within 650 nm to 700 nm against the absorbance over 675 nm to 685 nm. Error bars denote the standard error for *n* = 3 per species

The *V*/*I* maxima and minima versus the absorbance are shown in Fig. 8. A slight correlation is visible between the maximum and minimum magnitude of the *V*/*I* bands within 650 nm to 700 nm and the absorbance over 675 nm to 685 nm. In general, the magnitude of the bands increases with increasing absorbance. Both *F. serratus* and *F. spiralis* show positive and negative bands with a very large magnitude well outside this trend. This is similar for the large negative band of *A. elatior*. On the other hand, mature *S. latissima* and *Porphyra* sp. have a relatively low circular polarizance.

## Discussion

**Fig. 9:**
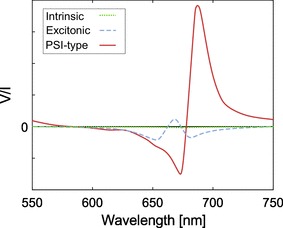
The three major sources of circular polarizance around the chlorophyll absorbance band in the red for higher plants for identical chlorophyll concentrations. Adapted after (Garab and van Amerongen 2009)

Different eukaryotic phototrophic organisms display different circular polarization spectra, with signal magnitudes that can vary by two orders of magnitude. Chlorophyll *a* itself exhibits a very weak intrinsic circular polarizance around 680 nm (Garab and van Amerongen 2009). Excitonic coupling between chlorophylls leads to a much larger signal in phototrophic bacteria and certain algae. In many more developed phototrophic organisms, the polarization spectra are dominated by the density and handedness of the supramolecular structures (psi-type circular dichroism), although these signals are superimposed on each other. Thus, for identical chlorophyll concentrations, the polarimetric spectral characteristics can vastly differ depending on the organization (see also Fig. 9).

The typical psi-type circular spectropolarimetric signals observed in vegetation are the result of the superposition of two relatively independent signals resulting from different chiral macrodomains in the chloroplast (Garab et al. 1988b, c, 1991a; Finzi et al. 1989). These psi-type bands of opposite sign do not have the same spectral shape and thus do not cancel each other out completely. The negative band is predominantly associated with the stacking of the thylakoid membranes, whereas the positive band mainly derives from the lateral organization of the chiral macrodomains formed by the PSII–LHCII complexes (Cseh et al. 2000; Dobrikova et al. 2003; Jajoo et al. 2012; Garab et al. 1991a).

Plant chloroplasts generally show little variation in structure (Staehelin 1986), which is noticeable in the circular polarization spectra of most plants (e.g., see the spectra of *Skimmia* and *Prunus* in Fig. 3). It has been reported before that the cpDNA sequences are extraordinarily conserved among plants and nearly identical in ferns, gymnosperms, and angiosperms (Palmer and Stein 1986). Of course, certain plants contain more chloroplasts per cell, or contain chloroplasts which are significantly larger or smaller, but in both cases, the normalized circular polarization will remain the same.

The polarimetric spectra of *Aspidistra* (Fig. 3) show a remarkably intense negative band, unlike the results usually encountered in plants. The positive band, however, has a magnitude that can be expected based on the lower absorbance as compared to the other higher plants we measured (see also Fig. 8). It has been shown that the contribution of both the negative and the positive band is dependent on the alignment of the chloroplasts (Garab et al. 1988c, 1991a), which might locally be aligned in such a way that only a single band dominates [e.g., near the veins of leaves (Patty et al. 2018b)]. The polarimetric spectra of *Aspidistra*, however, can be very well explained by the unusually large grana. Previous electron microscopy research on *Aspidistra elatior* chloroplasts revealed grana containing a vast number of thylakoid layers that may well exceed 100 (Steinmann and Sjöstrand 1955). As the positive and the negative bands overlap (leading to the split signal), it is to be expected that also the positive band is larger than encountered normally.

Similar to higher plants, also green algae contain PSII–LHCII supercomplexes utilized in photosynthesis. Between green algae and higher plants there are slight differences in the trimeric LHCII proteins and their isoforms, and, in addition, the green algae lack one of three minor monomeric LHCII polypeptides (CP24) [see also (Minagawa 2013) and references therein]. The green algae we measured show a spectral polarimetric profile that appears very similar to that of plants. However, the negative band centered around 650 nm is likely an excitonic band resulting from short-range interactions of the chlorophylls and the negative, usually stronger, psi-type band around 675 nm is virtually absent. The positive psi-type centered around 690 nm, on the other hand, is still present.

These results are unlike those reported for the unicellular green algae *Chlamydomonas reinhardtii*, which display a negative excitonic and a negative psi-type band of equal strength [e.g., see (Nagy et al. 2014)]. Importantly, the PSII–LHCII supercomplexes are far less stable in green algae as compared to plants, and it has been indicated that the L trimer (as well as the M and S trimers) could dissociate easily from PSII (Tokutsu et al. 2012). It has been shown that in *Ulva* flattening of the chloroplasts occurs under illumination, which additionally results in a decrease in thickness of the thylakoid membrane itself (Murakami and Packer 1970). Such fundamental changes in molecular structure might easily lead to (partial) dissociation of trimer L, which in turn can lead to the observed apparent absence of the negative psi-type band.

The red algae contain a more primitive photosynthetic apparatus that represents a transition between cyanobacteria and the chloroplasts of other algae and plants. This is also very evident from the displayed spectra in Fig. 5. For both species, the magnitude of the signal is small and comparable, even though *Porphyra* sp. had a much larger absorbance (Fig. 8), but the spectral shape suggests very fundamental differences in molecular structure. Surprisingly, *Porphyra* sp. shows a circular polarization spectrum with bands that might be associated with psi-type circular polarizance [at 675 nm (−) and at 690 nm (+)]. The origin and significance of these signals, however, require further investigation. The circular polarimetric spectra of *G. turuturu* lack these features but show two bands that can be associated with the excitonic circular polarization bands similar to those in cyanobacteria [at 670 nm (+) and at 685 nm (−)] [cf. (Sparks et al. 2009a)], which for a large part result from the excitonic interactions in PSI (Schlodder et al. 2007). In both species, the features between 550 and 600 nm might be associated with R-phycoerythrin (Bekasova et al. 2013). Additionally, in *Porphyra* sp., the sharp feature around 635 nm can be associated with phycocyanin (Sparks et al. 2009a). Both pigment–protein complexes belong to the phycobilisomes, which only occur in red algae and cyanobacteria and function as light-harvesting antennae for PSII while LHC is limited to PSI.

As in red algae and green algae, the brown algae contain no true grana but the thylakoid membranes are stacked in groups of three (Berkaloff et al. 1983). The brown algae measured in this study additionally contain chlorophyll *c*, which is slightly blue-shifted compared to chlorophyll *a* or *b*. Compared to chlorophyll *a*, chlorophyll *c*, however, has only a very weak contribution to the overall circular polarizance. Additionally, in brown algae, the light-harvesting antennae are homogeneously distributed along the thylakoid membranes (De Martino et al. 2000; Büchel and Garab 1997).

Interestingly, the juvenile *Saccharina* displays only a very weak negative band around 683 nm (Fig. 6). These results closely resemble those of isolated brown algae LHCs, which exhibit no excitonic bands but show solely a negative band around 680 nm. This band likely results from an intrinsic induced chirality of the chlorophyll *a* protein complex (Büchel and Garab 1997). The polarimetric spectra of mature *Saccharina* and *Undaria* show a split signal that is similar to that of higher plants. While the molecular architecture of the LHCs is very different from those in higher plants, the pigment–protein complexes in brown algae are organized in large chiral domains which give similar psi-type signals in circular polarizance (Szabó et al. 2008; Nagy et al. 2012). These intrinsic so-called fucoxanthin chlorophyll *a/c* binding proteins show a high homology to LHC in higher plants and have been shown to form complexes with trimers or higher oligomers (Lepetit et al. 2007; Büchel 2003; Katoh et al. 1989).

As shown in Fig. 7, the measured species of the genus *Fucus* exhibit an unusually large signal in circular polarizance, while the absorbance of the samples was within the range of the samples of the other species (Fig. 8). Although their spectral shapes are very similar to those of diatoms [cf. (Ghazaryan et al. 2016; Szabó et al. 2008; Büchel and Garab 1997)], the bands are two orders of magnitude stronger in *Fucus*. Most research on chlorophyll *a/c* photosynthesis is, however, carried out on diatoms and the reported size of the protein complexes again varies. Signals of such magnitude suggest that these macromolecular assemblies are much larger in *Fucus* than previously reported for other brown algae. Additionally, in the spectra of *Fucus*, a positive band can be observed around 595 nm. Most likely, this band and the weaker negative band around 625 nm can be assigned to chlorophyll *c*.

The results here show that the molecular and macromolecular organization of the photosynthetic machinery in algae is much more flexible and dynamic than reported before, likely due to larger inter-specific differences than generally assumed. Additionally, this also appears to be the case for one of the plants we measured (*A. elatior*), which displayed a negative psi-band one order of magnitude larger than ordinarily observed for higher plants.

When it comes to circular polarizance as a biosignature, it is important to note that efficient photosynthesis is not necessarily accompanied by large signals in circular polarization. While the intrinsic circular polarizance of chlorophyll is very low, the magnitude of the signals becomes greatly enhanced by a larger organization resulting in excitonic circular polarizance and ultimately psi-type circular polarizance. For the latter, the chiral organization of the macrodomains of the pigment–protein complexes is of importance, but it should be noted that the density of the complexes needs to be large enough (that is, significant coupling over the macrodomain is required) in order to function as a chiral macrodomain (Keller and Bustamante 1986). Many organisms thus display only excitonic circular polarizance, as is the case for certain algae measured in this study and generally bacteria. When psi-type circular polarizance is possible, the signals can easily become very large, in our study up to 2% for brown algae in transmission.

### Conclusions

We have measured the polarizance of various multicellular algae representing different evolutionary stages of eukaryotic photosynthesis. We have shown that the chiral organization of the macrodomains can vary greatly between these species. Future studies using molecular techniques to further characterize and isolate the complexes in these organisms are highly recommended. It will additionally prove very interesting to investigate these chloroplasts (including those with larger grana such as *Aspidistra*) using polarization microscopy (e.g., Steinbach et al. 2014; Finzi et al. 1989; Gombos et al. 2008). The high-quality spectra in this study and their reproducibility underline the possibility of utilizing polarization spectroscopy as a quantitative tool for non-destructively probing the molecular architecture in vivo.

Our results not only show variations in spectral shapes, but also in magnitude. Especially, the brown algae show a large variation, which is up to three orders of magnitude for the species measured in this study. Additionally, the induced fractional circular polarization by members of the genus *Fucus* is much larger than observed in vegetation. Future studies on the supramolecular organization in this genus and the variability caused by, for instance, light conditions, will also clarify the maximum extent of the circular polarizance by oxygenic photosynthetic organisms.

While the displayed results were obtained in transmission, the spectral features are also present in reflection. As such, future use of circular spectropolarimetry in satellite or airborne remote sensing could not only aid in detecting the presence of floating multicellular algae but also aid in species differentiation, which is important in regional biogeochemistry (Dierssen et al. 2015).

Importantly, while the presence of similar circular polarization signals is an unambiguous indicator for the presence of life, life might also flourish on a planetary surface and still show minimal circular polarizance (which for instance would have been the case on Earth if terrestrial vegetation evolved through different Archaeplastida/SAR supergroup lineages). On the other hand, these signals might also be much larger than we would observe from an Earth disk-averaged spectrum (which is the unresolved and therefore spatially integrated spectrum of a planet).

## Footnotes

1 Any mention of commercial products or companies within this paper is for information only; it does not imply recommendation or endorsement by the authors or their affiliated institutions.
